# Comparative functional expression of nAChR subtypes in rodent DRG neurons

**DOI:** 10.3389/fncel.2013.00225

**Published:** 2013-11-28

**Authors:** Nathan J. Smith, Arik J. Hone, Tosifa Memon, Simon Bossi, Thomas E. Smith, J. Michael McIntosh, Baldomero M. Olivera, Russell W. Teichert

**Affiliations:** ^1^Department of Biology, University of UtahSalt Lake City, UT, USA; ^2^Interdepartmental Neuroscience Program, University of UtahSalt Lake City, UT, USA; ^3^Department of Psychiatry, University of UtahSalt Lake City, UT, USA; ^4^George E. Wahlen Veterans Affairs Medical CenterSalt Lake City, UT, USA

**Keywords:** calcium imaging, DRG, nAChR, conotoxin, sensory neuron, neuronal subclass

## Abstract

We investigated the functional expression of nicotinic acetylcholine receptors (nAChRs) in heterogeneous populations of dissociated rat and mouse lumbar dorsal root ganglion (DRG) neurons by calcium imaging. By this experimental approach, it is possible to investigate the functional expression of multiple receptor and ion-channel subtypes across more than 100 neuronal and glial cells simultaneously. Based on nAChR expression, DRG neurons could be divided into four subclasses: (1) neurons that express predominantly α3β4 and α6β4 nAChRs; (2) neurons that express predominantly α7 nAChRs; (3) neurons that express a combination of α3β4/α6β4 and α7 nAChRs; and (4) neurons that do not express nAChRs. In this comparative study, the same four neuronal subclasses were observed in mouse and rat DRG. However, the expression frequency differed between species: substantially more rat DRG neurons were in the first three subclasses than mouse DRG neurons, at all developmental time points tested in our study. Approximately 70–80% of rat DRG neurons expressed functional nAChRs, in contrast to only ~15–30% of mouse DRG neurons. Our study also demonstrated functional coupling between nAChRs, voltage-gated calcium channels, and mitochondrial Ca^2^^+^ transport in discrete subsets of DRG neurons. In contrast to the expression of nAChRs in DRG neurons, we demonstrated that a subset of non-neuronal DRG cells expressed muscarinic acetylcholine receptors and not nAChRs. The general approach to comparative cellular neurobiology outlined in this paper has the potential to better integrate molecular and systems neuroscience by uncovering the spectrum of neuronal subclasses present in a given cell population and the functionally integrated signaling components expressed in each subclass.

## INTRODUCTION

Progress in investigating the mammalian nervous system has largely been achieved by molecular and systems neuroscientists at two very disparate levels. In between the molecular and systems levels, there is a forbidding complexity that arises from the many functionally divergent subclasses of neurons present at each anatomical locus. The complexity of cellular function is created, in part, by the differences in expression of many individual genes, across even closely related neuronal subclasses. Functional complexity is also created because individual gene products can combine to form various heteromeric receptor- and ion-channel subtypes in different neuronal subclasses. These factors create the functionally divergent neurons with distinct physiological roles found at any anatomical locus of the nervous system.

The molecular complexity is exemplified by nicotinic acetylcholine receptors (nAChRs), a family of ligand-gated ion channels. Functional nAChRs are pentameric, with 16 genes (in mammals) encoding different nAChR subunits. Because the subunits may be assembled into various combinations of heteromeric or (in some cases) homomeric combinations to form the functional pentameric ion channel, an enormous number of potential nAChR receptor subtypes are possible, with each combination that generates a functional receptor having a potentially distinct physiological function. The conventional approach to understand gene function, through targeted gene deletions (i.e., mutations in knockout mice or other model organisms), has limited value in this situation: ablation of a specific gene does not just result in the loss of function of one nAChR subtype, but of all functional nAChRs containing the subunit encoded by that gene. For example, if the β4 subunit is knocked out, then every functional nAChR that contains a β4 subunit (e.g., α6β4; α3β4; α6α3β4; α4β4; α6β4β2, etc.) would be functionally knocked out. This fact highlights the need for a pharmacological approach to selectively perturb the function of specific nAChR subtypes, in order to elucidate the physiological roles of each.

In this report, we have employed an approach to subdivide large, heterogeneous neuronal populations into subclasses by their functional expression of specific receptor- or ion-channel subtypes. Such neuronal subclasses (defined by expression of a single gene) could eventually be further subdivided into specific neuronal cell types (defined by a particular physiological function and expression of a cell-specific combination of genes), by cross-correlating the functional expression of several different genes across many individual neurons. The basic strategy is to monitor functional activity of specific receptor- and ion-channel subtypes in more than 100 individual cells simultaneously from a heterogeneous cell population, as we described previously (Teichert et al., 2012a,b). Using this experimental approach, we characterized dissociated rat and mouse lumbar dorsal-root ganglion (DRG) neurons that express functional nAChRs, and identified the particular nAChR subtypes expressed in different neuronal subclasses. In principle, this approach will allow us to investigate the function of a specific nAChR subtype in a specific neuronal subclass. A long-term goal of our laboratories is to understand the various roles of different nAChR subtypes expressed in different somatosensory neurons. For example, we seek to understand the roles of α7 or α3β4 nAChRs in modulating different sensory modalities (e.g., sensations of pain, itch, temperature or touch) in the various neuronal subclasses responsible for transmitting these signals from the periphery to the central nervous system. This work represents a critical first step toward that goal.

## MATERIALS AND METHODS

### PREPARATION OF SOLUTIONS

The medium for culturing DRG neurons, “MEM + supplements,” was as follows: minimal essential media [MEM, from Invitrogen (Life Technologies)], was supplemented with 10% fetal bovine serum (FBS, from Hyclone), penicillin (100 U/mL), streptomycin (100 μg/mL), 1× Glutamax (from Invitrogen), 10 mM HEPES, and 0.4% (w/v) glucose. The medium was adjusted to a pH of 7.4 with NaOH, filtered through a 0.22 μm filter under sterile conditions, and stored at 4°C until shortly before use, when it was allowed to warm to 37°C in a tissue-culture incubator with 5% CO_2_ atmosphere.

The “observation solution” (bath solution) for calcium-imaging experiments consisted of (in millimolar): 145 NaCl, 5 KCl, 2 CaCl_2_, 1 MgCl_2_, 1 sodium citrate, 10 HEPES, and 10 glucose. A 10× stock of observation solution was prepared with penicillin-streptomycin at 100 U/mL and 100 μg/mL, respectively and stored at 4°C. No additional penicillin-streptomycin was added to the 1× observation solution. Sodium citrate and glucose were added to the 1× solution to yield their final concentrations given above. The 1× solution was adjusted to a pH of 7.4 with NaOH and stored at 4°C until used at room temperature.

Hank’s balanced salt solution (HBSS), HEPES, and 2.5% trypsin were purchased from Invitrogen. DNase (II) type-I, collagenase-A, and acetylcholine chloride (ACh) were purchased from Sigma Aldrich. ω-Conotoxin GVIA, and ω-conotoxin MVIIC were purchased from Tocris Biosciences. All α-conotoxins were synthesized as previously described (Cartier et al., 1996). All stock solutions of pharmacological agents were diluted into observation solution to yield their final working concentrations described in Results and in the figures. Stock solutions were as follows: 1 M ACh in water; 200 μM α-conotoxin ArIB[V11L;V16D] in observation solution; 50 μM α-conotoxin AuIB in observation solution; 100 μM α-conotoxin BuIA in observation solution; 10 μM α-conotoxin BuIA[T5A; P6O] in observation solution; 200 μM ω-conotoxins GVIA and MVIIC in observation solution; 10 mM Nicardipine HCl in DMSO; Stock solutions of 100 mM PNU-120596 were prepared in DMSO and then completely diluted in one step into observation solution at 50°C while stirring to make 1 or 5 μM working concentrations. Fura-2-acetoxymethyl ester (Fura-2-AM, from Invitrogen) was dissolved in DMSO to produce a 1 mM stock solution, which was distributed into single-use aliquots and stored at -20°C. Fura-2-AM was used at 2.5 μM working concentration as described below.

### PREPARATION OF 24-WELL PLATES

Silicone rings were cut with cork borers from 0.5 or 0.25 mm thick silicone sheets (Grace BioLabs). Each ring had an outer diameter of ~14 mm and an inner diameter of ~4 mm. The rings were washed sequentially with 70% ethanol, deionized filtered water, 100, and 70% ethanol. They were then autoclaved and dried. Each silicone ring was placed on the floor of a well of a poly-D-lysine coated 24-well tissue-culture plate (BD Biosciences) and sealed to the floor with gentle pressure applied with the tips of a pair of dull forceps. The exterior wells were not used for cultures; instead those wells (without silicone rings) and spaces between wells were half-filled with sterile distilled water to humidify the atmosphere above the plate.

For some experiments, the plate floor in the center of each silicone ring was coated with mouse laminin (BD Biosciences) by applying 30 μL of 10 μg/mL laminin, dissolved in Hank’s Balanced Saline Solution (HBSS). Laminin was not used for experiments that directly compared mouse and rat DRG cells. The plates were then placed in a 37°C tissue-culture incubator for ~2 h to allow adequate time for laminin to coat the floor of each well. The laminin solution was replaced with MEM + supplements just prior to plating cells. This was done by aspirating the laminin solution and immediately adding 30 μL of MEM + supplements. Cells in suspension were added to the center of each silicone ring, onto the floor of the well, in the same manner (described in more detail below).

### PREPARATION OF DRG CELLS

All procedures complied with the rules and regulations in the National Institutes of Health *Guide for the Care and Use of Laboratory Animals *and were approved by the Institutional Animal Care and Use Committee (IACUC) of the University of Utah Health Sciences Center. In all cases, rats used were Sprague–Dawley and mice used were C57BL/6.

Several of the initial preparations of rat DRG cells were conducted as follows. After the rats were sacrificed with CO_2_, the lumbar dorsal root ganglia (L1-L6) were removed and placed in ice cold HBSS buffered with 10 mM HEPES, pH 7.2 (dissection buffer). The nerve roots were trimmed and the ganglia were then bisected or quartered, after which they were transferred to a 15 ml conical tube with 2 mL of dissection buffer containing 0.1% (wt/vol) collagenase-A and 0.25% (wt/vol) trypsin. The ganglia were incubated at 37°C for 60 min in the dissection solution containing enzymes, rinsed once with dissection solution, and then mechanically dissociated by trituration through a series of Pasteur pipettes of decreasing tip diameter (prepared by heating the tip in a flame while rotating the pipette) in solution containing 5 mM MgCl_2_and 10 μg/mL DNase (II) type-I. The volume of the cell suspension was increased to 10 mL with dissection solution and then passed through a 70 μm cell strainer to remove large pieces of tissue. At this point, the cells were collected by centrifugation at 200 × *g* for 2 min, after which the supernatant was removed by aspiration and the cells were resuspended in MEM + supplements at a volume (typically ~270 μL) and density suitable for plating cells in the previously prepared 24-well plates.

For experiments used to compare mouse and rat DRG cells, the following methods were applied consistently in preparing cell cultures so as to avoid any apparent differences that may arise from differences in methodology *per se*. In these cases, the 24-well plates were not coated with laminin, and no growth factors (i.e., GDNF) were added to the culture media. After a mouse or rat was sacrificed with CO_2_, the lumbar dorsal root ganglia were removed, and placed in ice cold HBSS without calcium or magnesium (Invitrogen). The nerve roots were trimmed and the ganglia were then bisected, after which they were transferred to a 15 ml conical tube with 1 mL of 0.25% (wt/vol) trypsin in HBSS without calcium or magnesium. The tube was then placed in a water bath at 37°C for 18 min. Following this incubation, 5 mL of pre-warmed (to 37°C) MEM + supplements were added to the solution and the DRG fragments were collected by centrifugation at 50 × *g* for 1 min. DRG fragments were resuspended in 1 or 2 mL of MEM + supplements. Cells were then mechanically dissociated by trituration through a series of Pasteur pipettes of decreasing tip diameter (prepared by heating the tip in a flame while rotating the pipette). Following mechanical dissociation, the cell suspension was passed through a 70 μm cell strainer to remove large pieces of tissue. Cells were collected by centrifugation at 50 × *g* for 5 min, after which the supernatant was removed by aspiration and the cells were then resuspended in MEM + supplements by gentle trituration with a 1 mL disposable plastic pipette tip. Cells were resuspended at a volume (typically ~270 μL) and density suitable for plating cells in the previously prepared 24-well plates.

### CELL CULTURE

Typically, 30 μL of the cell suspension was then added to the center of the silicone ring in each well of a 24-well plate, which was previously prepared as described above. Each 24-well plate was then placed in the 37°C, 5% CO_2_ tissue-culture incubator for 45–60 min to allow cells to settle and adhere to the floor of the pate within the silicone ring. Following this incubation period, 1 mL of pre-warmed (37°C) MEM + supplements was added very gently at the edge of each well to avoid dislocating any loosely adherent cells within the silicone ring. For some experiments, glial derived neurotrophic factor (GDNF) from PeproTech was added to MEM + supplements at a final concentration of 20 ng/mL. GDNF was not added to MEM + supplements for experiments used to compare mouse and rat DRG cells. Immediately following the addition of MEM + supplements, each plate was then returned to the 37°C, 5% CO_2_ tissue-culture incubator, and the cultures were used for imaging after 16–36 h.

### LOADING CELLS WITH FURA-2-AM

After culturing the cells overnight, the 24-well plate was placed in a sterile tissue-culture hood. The 1 mL of MEM + supplements in each well was agitated by pipetting it up and down vigorously in the well to suspend all dead cells and dislodge any cells that were only loosely adherent. In general, the remaining adherent cells were viable DRG neurons and glia. The medium was replaced with 500 μL of fresh MEM + supplements (without FBS) also containing 2.5 μM Fura-2-AM, which was freshly prepared by thawing the single-use stock aliquot of 1 mM Fura-2-AM in DMSO and adding it to MEM + supplements (without FBS), followed by vigorous vortexing for ~20 s. The plate was placed in the 37°C incubator for 1 h and then at room temperature for 30 min to load the cells with Fura-2-AM dye, prior to calcium imaging. At this time, the media was replaced in each well with fresh MEM + supplements (at room temperature) without Fura-2. Just prior to imaging a particular well, MEM + supplements was replaced with observation solution (at room temperature) at least three times to completely remove free Fura-2-AM from the well.

### VIDEO MICROSCOPY

Images were obtained either with: (1) a 10× 0.5 NA objective on an inverted Nikon Diaphot 200 microscope or (2) with a 10× 0.4 NA objective on an inverted Olympus IX70 microscope with a reducing lens in front of the camera to image a larger field of view. With both microscopes, a Sutter Instruments Lambda LS light source (300-W Xenon arc lamp) fitted with a filter wheel and shutter (controlled by a Lambda 10-B Smart Shutter, Sutter Instruments), was used as the source of excitation light at 380 and 340 nm. Images were acquired with a Nikon Digital Sight DS-Qi1Mc camera and controller and Nikon NIS elements software.

After loading cells with Fura-2-AM, a 24-well plate was fastened to the microscope stage. A brightfield image of a single field of view was captured and used to select regions of interest (ROI) corresponding to the area (delineated by the outer perimeter) of all non-overlapping cells in the field. Each ROI, corresponding to a single cell, was monitored for changes in [Ca^2^^+^]_i_. Typically, ~100 neurons were imaged for each experiment. The fluorescence emission was monitored at 510 nm for both 380 and 340 nm excitation. The exposure time for resting cytosolic calcium levels was adjusted for each experiment to a maximum ROI intensity of ~3500 gray levels for 380 nm excitation and ~1000 gray levels for 340 nm excitation. An image was captured at each excitation wavelength and the ratio of fluorescence intensities at excitation wavelengths of 340 and 380 nm was acquired either once per second or once per 2 s to monitor the relative changes in calcium concentration in each cell as a function of time.

### EXPERIMENTAL PROTOCOLS

Calcium signals were elicited by a ~15-s application of 1 mM ACh (ACh pulse), in observation solution, as follows: the observation solution was aspirated from the well with a peristaltic pump controlled by a foot pedal, and observation solution containing 1 mM ACh was applied manually at the edge of the well from a pipette tip with a silicone tubing extension, the flexibility of the latter minimized any movement of the plate. After 15 s, the ACh solution was replaced completely with observation solution in the same manner. Typically, the observation solution was replaced three or four more times over the subsequent 45 s to remove any residual ACh from the well. This procedure was repeated as necessary, generally at intervals ranging from 5 to 8 min. In some cases, a high concentration of extracellular potassium (e.g., 30–50 mM [K^+^]_o_) was added to the bath at the beginning or end of each trial to help differentiate between neuronal and non-neuronal cells. Non-neuronal cells did not respond to depolarization by high [K^+^]_o_. Additionally, the somas of non-neuronal cells in these cultures are smaller in diameter than the somas of DRG neurons. A pulse of high [K^+^]_o_ was applied to the bath in the same manner as an ACh pulse described above, with identical washing procedures. The ~15-s applications of ACh or [K^+^]_o_ are represented in the figures by arrows at each respective time point, where the letter “A” represents ACh application and the letter “K” represents application of high [K^+^]_o_ . Horizontal bars in each figure represent the application of other pharmacological agents to the bath solution for the duration of the bar (on the time scale of the *X* axis), as described in figures and figure legends. The experimental protocol shown at the bottom of each figure (or panel) corresponds to all calcium-imaging traces shown in that figure (or panel).

### STATISTICAL DATA ANALYSIS

The statistical data analysis of capsaicin sensitivity across DRG neuronal subclasses was performed as follows. We conducted 10 independent experimental trials for mouse DRG and 8 independent experimental trials for rat DRG. For each independent experimental trial, we executed the same experimental protocol in a different well containing a mixed population of DRG cells. Only neurons that responded to depolarization by 30 mM [K^+^]_o_ were included in the data analysis. Neurons were parsed into different subclasses by functional nAChR expression as described in the Results. From each independent experimental trial, we calculated the percentage of capsaicin-sensitive DRG neurons within each neuronal subclass (sample means). These sample means were then compared to each other by single-factor Analysis of Variance (ANOVA) using Microsoft Excel. Significant differences were set at *p*-value < 0.05.

## RESULTS

The experiments in this study were carried out with dissociated rat or mouse lumbar DRG neurons loaded with Fura-2 dye for calcium imaging. Several related experimental protocols were used to assess the functional expression of particular nAChR subtypes, as illustrated in the figures and as described in Materials and Methods. In general, pulses of 1 mM acetylcholine (ACh) were applied to a heterogeneous population of dissociated DRG neurons at regular time intervals to elicit transient increases in cytoplasmic-calcium concentration, [Ca^2+^]_i_, observed as peaks in the traces in each figure. Typically the responses of 50–150 neurons were imaged individually and simultaneously. ACh-elicited calcium signals observed prior to application of nAChR antagonists served as controls. ACh-elicited calcium signals observed following application of subtype-selective nAChR antagonists were used to identify the nAChR subtypes that were functionally expressed in each cell. The nAChR antagonists used in the experiments are summarized in **Table 1**. These are α-conotoxins with high selectivity for specific nAChR subtypes.

**Table 1:** IC_50_ values for inhibition of nAChR subtypes by various α-conotoxins and their analogs (adapted from Hone etal., 2012).

	α3β2	α3β4	α4β2	α4β4	α6/α3β2β3	α6β4	α7
ArIB[V11L;V16D]	>20 μM	>20 μM	>20 μM	>20 μM	>20 μM	>20 μM	1.1 nM
BuIA	5.7 nM	27.7 nM	>10 μM	69.9 nM	258 pM	1.5 nM	272 nM
BuIA[T5A;P6O]	>10 μM	1.2 μM	>10 μM	>10 μM	>10 μM	58.1 nM	>10 μM
AuIB	>100 μM	750 nM	>100 μM	>100 μM	>100 μM	7.3 μM	~10 μM

### IDENTIFICATION OF A RAT DRG NEURONAL SUBCLASS THAT FUNCTIONALLY EXPRESSES nACRs CONTAINING β4 SUBUNITS

**FIGURE 1:**
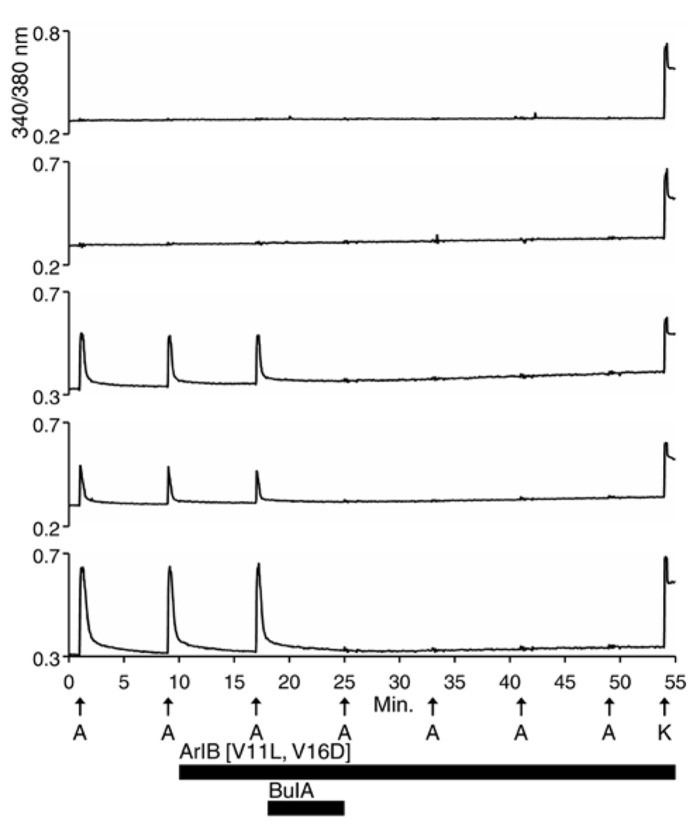
**Functional expression of nAChRs that contain β4 subunits in a subset of rat DRG neurons.** One mM ACh was applied to dissociated Rat DRG neurons at 8 min intervals as described in Section “Materials and Methods,” and as indicated by vertical arrows directly above the letter “A” (A = ACh). This abbreviation applies to all subsequent figures. At the end of the experiment, 50 mM [K^+^]_o_ (indicated by arrow above letter “K”) was applied to the cells to elicit a calcium signal through a membrane depolarization. Cells that did not respond to 50 mM [K^+^]_o_ were excluded from the analysis, because they were considered to be non-neuronal cells or non-viable neurons. The top two traces are from neurons that did not respond to ACh but did respond to 50 mM [K^+^]_o_. The bottom three traces are from neurons that responded to ACh and 50 mM [K^+^]_o_. At 10 min, after two ACh pulses, 200 nM ArIB[V11L;V16D], which selectively inhibits α7 nAChRs, was applied to the bath solution for the duration of the experiment, as indicated by a long horizontal bar. After the cells were exposed to ArIB[V11L;V16D] for 7 min, an ACh pulse was applied at 17 min. Very little, if any, block was observed after application of the α7-selective peptide. At 18 min, 10 μM BuIA was applied to the bath for 7 min as indicated by a short horizontal bar. This peptide is a broad-spectrum blocker of nAChRs. However, BuIA inhibition of most nAChRs is rapidly reversible; in contrast, it is very slowly reversible from nAChRs that contain β4 subunits. The apparently irreversible block of the ACh-responsive neurons by BuIA suggested that the predominant nAChR subtype in Rat DRG neurons is a β4-containing nAChR.

Each trace shown in **Figure 1** represents a single neuron’s response. In **Figure 1**, calcium-imaging traces from three ACh-responsive DRG neurons are shown (bottom three traces), and from two non-ACh-responsive neurons (top two traces), all from a single experimental trial (one field of view from a single well). Following the application of 200 nM α-conotoxin ArIB[V11L;V16D] (hereafter ArIB[V11L;V16D]), an nAChR antagonist selective for the α7 subtype (**Table 1**; Whiteaker et al., 2007), there was no obvious effect on the ACh response. In contrast, the addition of 10 μM α-conotoxin BuIA (BuIA), a broad-spectrum antagonist of nAChRs (**Table 1**; Azam et al., 2005), resulted in complete inhibition of the ACh response (**Figure 1**). BuIA blocks most nAChR subtypes with rapid reversibility, but its inhibition of nAChRs that contain a β4 subunit is very slowly reversible (Azam et al., 2005). The apparently irreversible block (over the time interval shown in **Figure 1**) suggested that a β4-containing nAChR was likely to be the predominant nAChR subtype in a subset of cultured rat DRG neurons.

### UNMASKING A RAT DRG NEURONAL SUBCLASS THAT FUNCTIONALLY EXPRESSES α7 nAChRs

**FIGURE 2:**
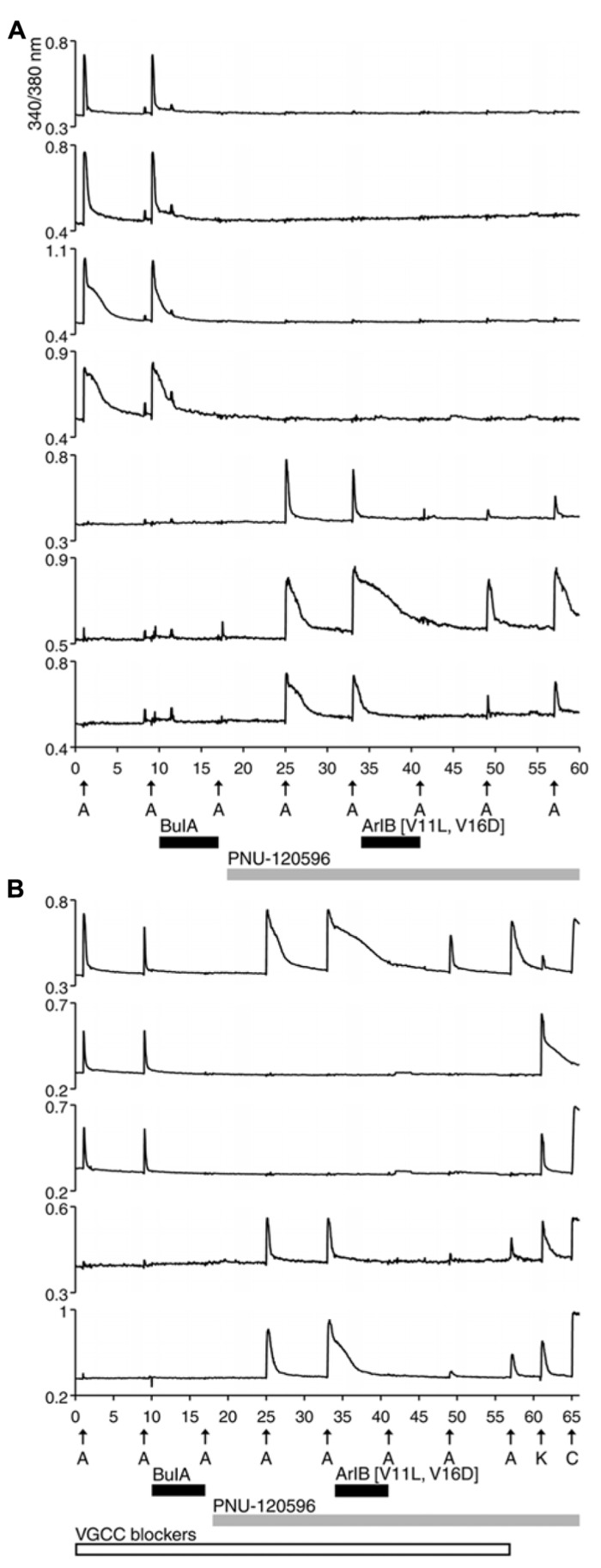
**Functional expression of α7 nAChRs and β4-containing nAChRs in different rat DRG neuronal subclasses.** After two ACh pulses,10 μM BuIA was applied to the bath as indicated by a short, black horizontal bar. The ACh-responsive neurons were blocked irreversibly by BuIA. After block by BuIA, 1 μM PNU-120596 (PNU) was applied to the bath solution for the duration of the experiment, as indicated by a long, gray horizontal bar. After application of PNU, ACh pulses revealed a second class of DRG neurons that were previously unresponsive to ACh in the absence of PNU. ACh responses in this second subclass of DRG neurons were blocked completely and reversibly by 200 nM ArIB[V11L;V16D]. **(A)** Traces of responses from selected neurons in an experimental trial conducted without VGCC blockers. **(B)** Traces of responses from selected neurons in an experimental trial conducted in the presence of VGCC blockers, as indicated by a long, open horizontal bar. The cocktail of VGCC blockers consisted of 200 nM ω-conotoxin GVIA to block N-type calcium channels, 200 nM ω-conotoxin MVIIC to block P/Q-type calcium channels, and 1 μM nicardipine to block L-type calcium channels. This protocol eliminated the shoulders observed for ACh-elicited calcium signals in the β4-expressing subclass, suggesting that those shoulders were caused by co-activation of nAChRs and VGCCs. This protocol also confirms that the signals observed in **(B)** are from calcium influx through nAChRs. At the end of this experiment, a pulse of 25 mM [K^+^]_o_ (K) was followed by application of 300 nM capsaicin (C), to demonstrate that these responses were from viable neurons (a subset of DRG neurons respond to capsaicin). The top trace demonstrates the response from an additional neuronal subclass that expresses both α7 nAChRs and β4-containing nAChRs.

Previous studies reported that α7 nAChRs are expressed in DRG neurons. This was demonstrated directly using standard electrophysiological techniques in a complementary study (Hone et al., 2012). However, using the protocol shown in **Figure 1**, there was no ACh response consistent with the α7 nAChR subtype. A possible reason for the failure to detect α7 nAChRs is that calcium transients elicited by the opening of α7 receptors are too small to be detectable using standard calcium-imaging methods, because of the rapid desensitization kinetics of α7 nAChRs. In order to increase the magnitude of change in [Ca^2+^]_i_, we applied a positive allosteric modulator specific for α7 receptors, PNU-120596 (PNU). Experimental results in the presence of PNU are shown in **Figure 2**.

In **Figure 2A**, four cells (top four traces) responded to ACh directly, prior to the addition of PNU. Consistent with the experiment in **Figure 1**, their ACh responses were inhibited irreversibly over the time frame of the experiment by the addition of 10 μM BuIA. After 1 μM PNU was added, some neurons that previously did not respond to ACh began to respond robustly to ACh application (**Figure 2A**, bottom three traces). All ACh responses that were elicited in the presence of PNU (following application of BuIA) were blocked by 200 nM ArIB[V11L;V16D]. As shown in **Figure 2A** (bottom three traces), a slow recovery of ACh responses was observed after washout of ArIB[V11L;V16D].

These experiments suggested that DRGs contained two major subclasses of ACh-responsive neurons. The first subclass appeared to express predominantly β4-containing nAChRs. These responded directly to ACh in the absence of PNU and their ACh responses were blocked by BuIA irreversibly over the time course of the experiment. The second subclass of DRG neurons appeared to express predominantly α7 nAChRs. These responded to ACh only in the presence of PNU and their ACh responses were blocked reversibly by ArIB[V11L;V16D]. In most cases, neurons that responded to ACh prior to application of PNU did not respond to ACh in the presence of PNU after BuIA was applied. Thus, the data suggest that ACh-responsive DRG neurons express predominantly either α7 nAChRs without substantial expression of β4-containing nAChRs, or β4-containing nAChRs without substantial α7 expression (**Table 2**).

**Table 2:** Percentages of the total population of mouse and rat DRG neurons that exhibited functional expression of different nAChR subtypes.

	Mouse			Rat		
	P14	P20–30	P42	P15–17	P30–40	P52
% α7+ only	11	7	14	20	35	27
% β4+ only	8	6	9	34	31	36
% α7+ and β4+	3	2	5	15	12	10
% ACh unresponsive	78	85	72	31	22	27

*“P” refers to the age of animals in postnatal days. For each age category of mouse or rat (each column in the table), the percentages of DRG neurons were calculated from ≤9 independent experimental trials that encompassed >600 total neurons (cells responsive to depolarization by high extracellular potassium concentration) in each case (each column).
*

### A DISTINCTIVE RAT DRG NEURONAL SUBCLASS THAT EXPRESSES BOTH α7 AND β4-CONTAINING nAChRs

We employed the protocol shown in **Figure 2B** multiple times in different trials, while simultaneously monitoring the responses from >50 individual DRG neurons in each trial. Notably, the experimental protocol in **Figure 2B** was done in the presence of a cocktail of voltage-gated calcium channel (VGCC) blockers, further supporting the evidence that the observed responses were mediated by nAChRs.

A minor fraction of the rat DRG neurons appeared to express both β4-containing nAChRs and α7 nAChRs (**Figure 2B**, top trace). Thus, the experiments establish four subclasses lumbar DRG neurons with respect to expression of nAChRs: (1) those that express predominantly β4-containing nAChRs, (2) those that express predominantly α7 nAChRs, (3) those that express both α7 and β4-containing nAChRs, and (4) those that do not respond to ACh in the presence or absence of PNU (**Table 2**).

### DRGs FROM MOUSE AND RAT INCLUDE THE SAME FOUR NEURONAL SUBCLASSES WITH RESPECT TO AChR EXPRESSION, BUT IN SUBSTANTIALLY DIFFERENT PROPORTIONS

**FIGURE 3:**
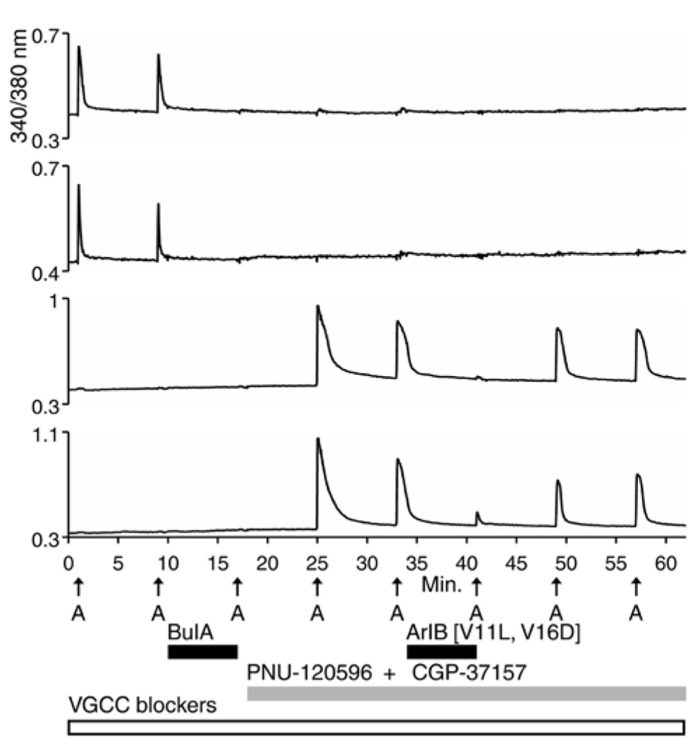
**Functional expression of α7 nAChRs and β4-containing nAChRs in different mouse DRG neuronal subclasses.** Like rat DRG neurons, mouse DRG neurons could be divided into subclasses that express either α7 nAChRs or β4-containing nAChRs. Shown are traces of responses from selected neurons in an experimental trial conducted in the presence of VGCC blockers, as indicated by a long, open horizontal bar. The cocktail of VGCC blockers consisted of 200 nM ω-conotoxin GVIA to block N-type calcium channels, 200 nM ω-conotoxin MVIIC to block P/Q-type calcium channels, and 1 μM nicardipine to block L-type calcium channels. This protocol eliminated the shoulders observed for ACh-elicited calcium signals in the β4-expressing subclass, suggesting that those shoulders were due to activation of VGCCs. This protocol also confirms that the signals observed are from calcium influx through nAChRs as was the case with rat DRG neurons also (see **Figure 2**). After two ACh pulses, 10 μM BuIA was applied to the bath as indicated by a short, black horizontal bar. The ACh-responsive neurons were blocked irreversibly by BuIA, as was the case with rat DRG neurons also (see **Figure 2**). After block by BuIA, 5 μM PNU-120596 (PNU) was applied to the bath solution for the duration of the experiment, as indicated by a long, gray horizontal bar. After application of PNU, ACh pulses revealed a second subclass of DRG neurons that were previously unresponsive to ACh, and that were not blocked irreversibly by BuIA. ACh responses in this second subclass of DRG neurons were blocked reversibly by 200 nM ArIB[V11L;V16D], similar to rat DRG neurons (see **Figure 2**). Because the broad shoulders of peaks persisted in the PNU-elicited ACh responses in the presence of VGCC blockers (see **Figure 2**), we applied PNU in combination with the blocker of the mitochondrial Na^+^/Ca^2^^+^ exchanger, CGP37157 (CGP) (10 μM), which eliminated the broad shoulders of ACh-elicited, PNU-amplified peaks.

**Figure 3** demonstrates that mouse DRG neurons include those that express predominantly α7 nAChRs (bottom two traces) and others that express predominantly β4-containing nAChRs (top two traces), just like rat DRG neurons (see **Figures 2** and **3**). The percentages of mouse and rat lumbar DRG neurons corresponding to each subclass, at different developmental time points, are summarized in **Table 2**. The same four neuronal subclasses, with respect to nAChR expression, were observed for both mouse and rat DRG. However, **Table 2** shows a conspicuous difference in the proportion of cells that express nAChRs in mouse and rat. Surprisingly, only ~15–30% of mouse DRG neurons responded to ACh (either in the presence or absence of PNU), in contrast to the ~70–80% of rat DRG neurons that responded to ACh, at all developmental time points tested. Thus, there is a clear difference in the frequency of DRG neurons responsive to ACh in the two species. Notably, only neurons responsive to depolarization by high extracellular potassium concentration were included in the data summary presented in **Table 2**.

### SPECIES DIFFERENCE IN CAPSAICIN SENSITIVITY AMONG NEURONAL SUBCLASSES

After obtaining the data for **Table 2**, we then investigated the capsaicin sensitivity of each neuronal subclass defined by nAChR expression from mature mouse and rat DRG. Capsaicin activates the TRPV1 channel and may demarcate nociceptive neurons (Caterina et al., 1997; Caterina and Julius, 2001). In mouse DRG, we did not observe significant differences in the percentages of capsaicin-sensitive neurons across neuronal subclasses defined by nAChR expression (**Table 3**, ANOVA *p*-value = 0.65). However, in rat DRG, we did observe significant differences in the capsaicin sensitivity of these neuronal subclasses (**Table 3**, ANOVA *p*-value < 0.001). These data suggest that in rat DRG the neuronal subclass that expresses only α7 nAChRs is significantly enriched for TRPV1 expression (94% of these neurons were capsaicin sensitive), whereas the neuronal subclass that expresses only β4-containing nAChRs included a significantly lower percentage of TRPV1-positive neurons (36%) than the other three neuronal subclasses (**Table 3**). Although the neuronal subclass that expresses both α7 and β4-containing nAChRs appears to be enriched for large-diameter neurons in both mouse and rat DRG (**Table 3**), it was evident that this neuronal subclass included both small- and large-diameter neurons (not shown).

**Table 3:** Average percentages of neurons responsive to 300 nM capsaicin and their average cross-sectional cell areas (cell size) within different mature DRG neuronal subclasses defined by functional nAChR expression.

	Mouse (>P40)		Rat (>P47)	
DRG neuronal subclass	Average % capsaicin sensitive	Average cell size (μm^2^)	Average % capsaicin sensitive	Average cell size (μm^2^)
α7+ only	44%	350	94%	396
β4+ only	56%	334	36%	410
α7+ & β4+	45%	392	79%	527
ACh unresponsive	43%	244	77%	380

*“P” refers to the age of animals in postnatal days. Mouse data was compiled from 10 independent experimental trials that encompassed >700 total neurons (cells responsive to depolarization by high extracellular potassium concentration). Rat data was compiled from eight independent experimental trials that encompassed >600 total neurons.*

### IDENTIFICATION OF α SUBUNITS PRESENT IN β4-CONTAINING nAChRs

**FIGURE 4:**
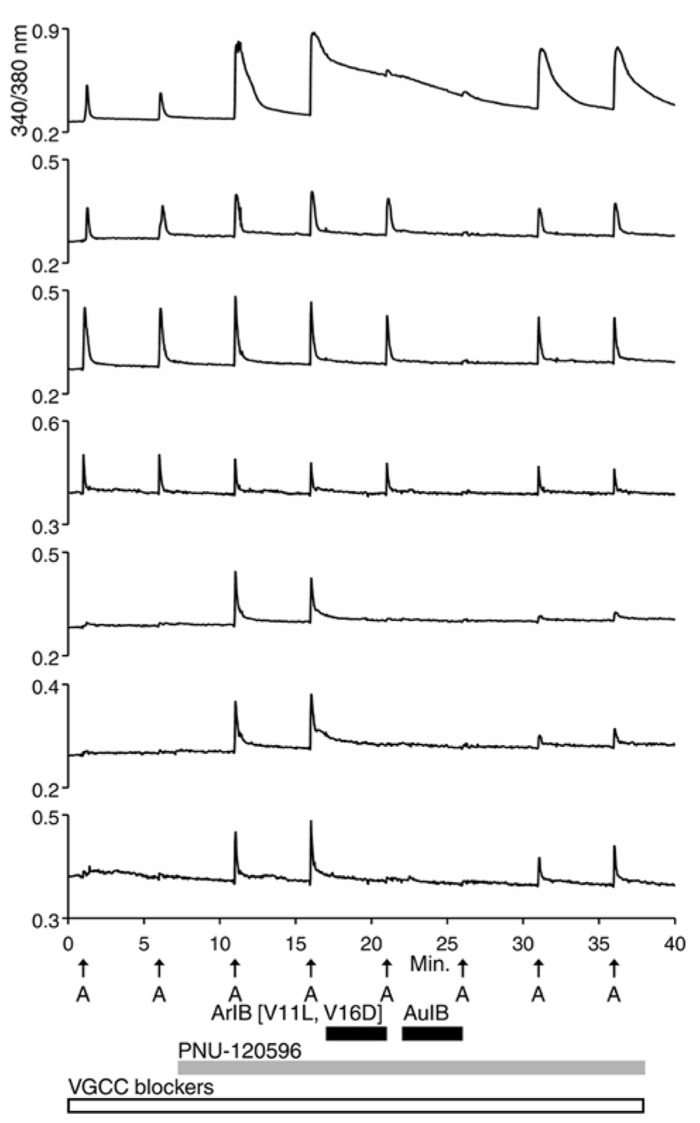
**The β4-containing nAChRs include α3 or α6 subunits.** This experiment was conducted with rat DRG neurons in the presence of a cocktail of VGCC blockers, as described in the **Figure 2** legend. PNU-120596 (1 μM) was applied after the second ACh pulse, revealing the α7-expressing subclass. ACh responses from the α7-expressing subclass were blocked reversibly by 200 nM ArIB[V11L;V16D] (bottom three traces), which did not block the neuronal subclass that expresses primarily β4-containing nAChRs. ACh responses from the β4-expressing nAChRs were blocked reversibly by 50 μM AuIB (second through fourth traces from the top), suggesting that the β4-expressing nAChRs also contain α3 or α6 subunits (see **Table 1**). Short, black horizontal bars indicate when the different conotoxins were present in the bath. The top trace illustrates a response from an additional neuronal subclass that expresses both α7 nAChRs and β4-containing nAChRs.

For the β4-expressing neurons, we wanted to identify the α subunits co-expressed with β4 to form functional ion channels. The experiment shown in **Figure 4**, using rat DRG neurons, was carried out with α-conotoxin AuIB (AuIB), which selectively inhibits α3β4 and α6β4 nAChRs over α4β4 and α4β2 nAChRs (**Table 1**; Luo et al., 1998). If the ACh-responsive cells were inhibited by 50 μM AuIB, this would suggest that the nAChR contained α3 or α6 subunits, but if they were not inhibited by AuIB, the α4 subunit might be present.

The experiment shown in **Figure 4** demonstrates that the application of 50 μM AuIB did indeed inhibit the ACh responses in the β4-expressing subclass (top four traces). However, in contrast to the effect of BuIA, the inhibition was readily reversible upon washing, as expected for block of α3β4 and α6β4 nAChRs by AuIB. The reversible inhibition by AuIB, and the irreversible inhibition by BuIA, cumulatively suggest that the predominant β4-containing subtypes of nicotinic receptors present in DRG neurons are either α3β4 or α6β4 (or a combination of both subtypes). Notably, **Figure 4** (top trace) also demonstrates that the subclass expressing both α3β4/α6β4 and α7 nAChRs can be detected with a different experimental protocol than the one employed in **Figure 2B** (top trace).

**FIGURE 5:**
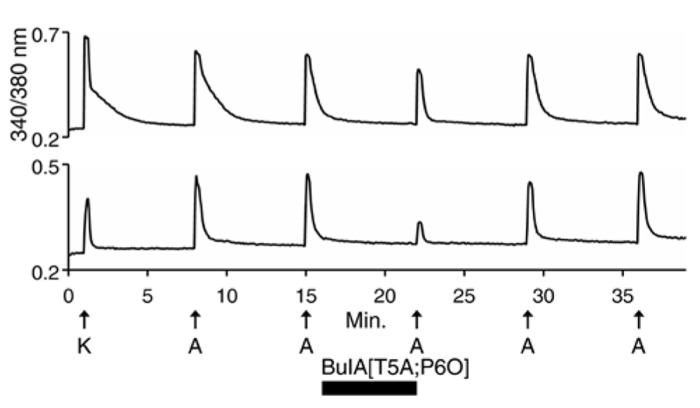
**Variable combinations of α6β4 and α3β4 nAChRs are expressed together. The two traces shown are from mouse DRG neurons.** Similar results were obtained with rat DRG neurons. In all of the β4-expressing neurons, responses to ACh were partially blocked by 500 nM BuIA[T5A;P6O], which selectively blocks α6β4 over α3β4 nAChRs (see **Table 1**). The bottom trace illustrates greater block by BuIA[T5A;P6O] than the top trace.

We then used 500 nM α-conotoxin BuIA[T5A;P6O] (hereafter BuIA[T5A;P6O]) to selectively block α6β4 over α3β4 nAChRs (**Table 1**; Azam et al., 2010). BuIA[T5A;P6O] partially blocked ACh-elicited responses from β4-expressing mouse DRG neurons (**Figure 5**). Similar results were obtained with rat DRG neurons, suggesting that α6β4 nAChRs mediate a portion of the ACh-elicited calcium signal in these neurons. However, the partial block by 500 nM BuIA[T5A;P6O] also suggested that there is another nAChR subtype contributing to the ACh-elicited responses. The complete block of ACh-elicited responses by 50 μM AuIB (**Figure 4**) suggested that the second component is mediated by α3β4 nAChRs. Although there is no concentration of BuIA[T5A;P6O] that is completely selective for α6β4 over α3β4 nAChRs (**Table 1**), it was apparent that the mix of α3β4 and α6β4 varied between cells (**Figure 5**). All ACh responses elicited in the absence of PNU were blocked in varying degrees by BuIA[T5A;P6O]. Greater block suggested higher expression of α6β4 relative to α3β4.

### SHOULDERS OF CALCIUM TRANSIENTS MEDIATED BY VOLTAGE-GATED CALCIUM CHANNELS AND MITOCHONDRIAL Na^+^/Ca^2+^ EXCHANGE

In the experiment shown in **Figure 2A**, considerable variability was observed in the decay kinetics of the ACh-elicited calcium signals in different neurons. Following a pulse of ACh, in some neurons [Ca^2^^+^]_i_ returned to baseline relatively rapidly (sharp peaks, e.g., top trace), while in other neurons [Ca^2^^+^]_i_ returned to baseline slowly (broad peaks, e.g., fourth trace from top), often with characteristic shoulders observed on the peaks. As shown in the top three traces of **Figure 2B**, the peaks were notably sharpened in cells that responded to ACh prior to application of PNU, when a cocktail of VGCC inhibitors was present in the bath solution. The variation in the shape of the peaks apparently was caused (in the absence of PNU) by a membrane depolarization through nAChR activation that was sufficient to trigger the activation of VGCCs to variable extents.

In some cases, the broad shoulders of the peaks mediated by α7 nAChR activation (with PNU) persisted in the presence of VGCC blockers (**Figures 2** and **4**). In these cases, the response profile hypothetically may be due to the release of calcium from internal calcium stores, triggered by the opening of the α7 nAChRs. With VGCC blockers in the bath solution, such ACh-response profiles were only observed in the α7-nAChR-expressing neurons in the presence of PNU. In DRG neurons, shoulders of calcium transients elicited by membrane depolarization were previously shown to be mediated by a mitochondrial Na^+^/Ca^2^^+^ exchanger (NCX; Baron and Thayer, 1997;Castaldo et al., 2009). Upon strong depolarization, the increase in cytoplasmic Ca^2^^+^ mediated by influx of Ca^2^^+^ through voltage-gated Ca^2^^+^ channels is modulated by concurrent Ca^2^^+^ influx into the mitochondria via a Ca^2^^+^ uniporter. After repolarization, mitochondrial Ca^2^^+^ is transported back into the cytoplasm via the mitochondrial Na^+^/Ca^2^^+^ exchanger (NCX), where it binds Fura-2, thus prolonging the observed calcium transient and creating a shoulder on the peak (Baron and Thayer, 1997; Castaldo et al., 2009). The mitochondrial Na^+^/Ca^2^^+^ exchanger (NCX) can be blocked by CGP37157 (CGP; Baron and Thayer, 1997). In the α7-expressing cells, the broad shoulders were eliminated when both VGCC blockers and CGP were co-applied with PNU (**Figure 3**, bottom two traces).

### ACh RESPONSES MEDIATED BY mAChRs IN NON-NEURONAL CELLS VS. nAChRs iN NEURONS

**FIGURE 6:**
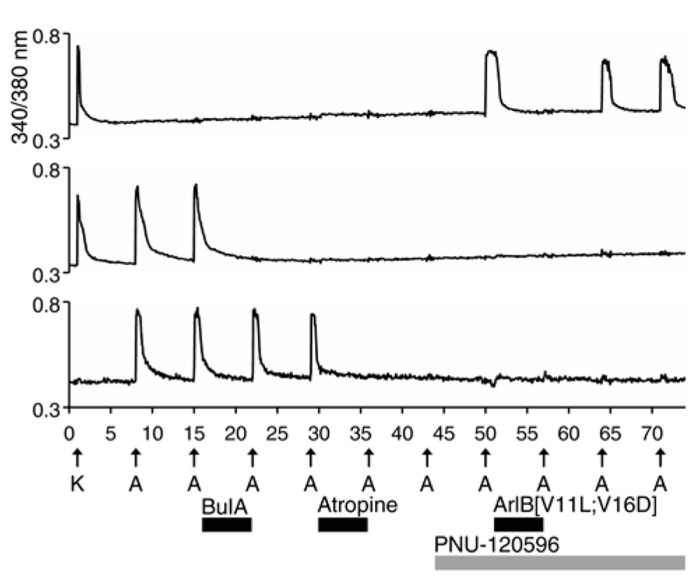
**A subset of non-neuronal DRG cells express muscarinic acetylcholine receptors (mAChRs).** The three traces shown are from mouse DRG cells. Similar results were obtained with rat DRG cells. The bottom trace is from a non-neuronal DRG cell that did not respond to depolarization by 30 mM [K^+^]_o_ (K), but did respond to applications of 1 mM ACh. Notably, the ACh-elicited responses in this cell were not blocked by BuIA, but were blocked by 1 μM atropine, an antagonist of mAChRs. In contrast to the bottom trace, the top two traces are from DRG neurons that responded to depolarization by 30 mM [K^+^]_o_. The top trace is from the DRG neuronal subclass that expresses α7 nAChRs and the middle trace is from the α3β4/α6β4-expressing subclass.

All of the ACh-elicited responses observed in DRG neurons were mediated by nicotinic (nAChRs) and not muscarinic acetylcholine receptors (mAChRs), as demonstrated by the block of ACh-elicted responses with subtype-selective conotoxins (**Figures 1**–**5;** **Table 1**). However, a small subset of non-neuronal cells (identified by lack of response to depolarization and by cell diameters smaller than neurons) in the DRG cell population also responded to ACh with transient increases in [Ca^2^^+^]_i_. In some non-neuronal cells, such ACh-elicited responses were not blocked by 10 μM BuIA, but were blocked by 1 μM atropine, an antagonist of mAChRs (**Figure 6**, bottom trace). Thus, **Figure 6** illustrates that our experimental approach is useful for investigating a broad set of receptors and ion channels expressed in both neuronal and non-neuronal cell types.

## DISCUSSION

In this study, we characterized the spectrum of rat and mouse DRG neurons with regard to their nAChR-expression profiles, using a cellular neuropharmacological platform that we established previously (Teichert et al., 2012a,b). Using this approach, we can directly compare individual cellular responses from greater than 100 DRG neurons simultaneously in a single experimental trial (one field of view from a single well). From the data compiled, four broad categories of rodent DRG neurons could be defined with respect to expression of different nAChR subtypes: (1) neurons that express predominantly β4-containing nAChRs with α3 and α6 subunits; (2) neurons that express predominantly α7 nAChRs; (3) neurons that express a combination of α3β4/α6β4 and α7 nAChRs; and (4) neurons that do not express nAChRs. However, in rat, each of the first three neuronal subclasses (that express nAChRs) encompasses a higher percentage of the total DRG cell population than in mouse (**Table 2**). Approximately 70–80% of rat DRG neurons expressed functional nAChRs, whereas only ~15–30% of mouse DRG neurons expressed functional nAChRs, at all developmental time points tested in our study (**Table 2**).

In rat DRG, there were significant differences in the percentages of capsaicin-sensitive (TRPV1 expressing) neurons between nAChR-defined neuronal subclasses. Within the neuronal subclass that expresses only α7 nAChRs, 94% of the neurons were capsaicin sensitive, whereas only 36% of the neurons were capsaicin sensitive in the subclass that expresses only β4-containing nAChRs (**Table 3**). In contrast to rat DRG, there were no significant differences in the percentages of capsaicin-sensitive neurons among the mouse DRG neuronal subclasses.

Presently, we do not know why these apparent differences exist between rat and mouse DRG neurons, but the results suggest that the α7 subclass may be enriched for nociceptors in rat DRG, whereas the β4-containing subclass may be comprised largely of non-nociceptive neurons. It is notable that a higher percentage of rat DRG neurons expressed α3β4/α6β4 nAChRs, α7 nAChRs, and TRPV1 channels than mouse DRG neurons (Table 2 and 3). Although this TRPV1 difference (capsaicin sensitivity) between mouse and rat has been observed in other studies (Caterina et al., 2000; Tognetto et al., 2001; Haberberger et al., 2004; Hjerling-Leffler et al., 2007), the generally broader expression pattern of multiple ion channels among rat DRG neuronal subclasses requires further investigation in future comparative studies. We should also consider the possibility that other nAChR subtypes may not be expressed in the soma of DRG neurons used for calcium imaging in this study, but may be present in nerve endings, axons, or synaptic terminals. These possibilities may also be investigated in future studies.

In this study, all of the ACh responses observed in DRG neurons from mouse and rat were mediated by nAChRs and not mAChRs. This was demonstrated by the block of ACh-elicited responses in neurons by subtype-selective conotoxins. In contrast, ACh-elicted responses from some non-neuronal cells were mediated by mAChRs. This was demonstrated by block of ACh-elicited responses by atropine, and by the lack of block by BuIA.

We observed some differences in pharmacology between mouse and rat: mouse DRG neurons required a higher concentration of PNU (5 μM in mouse vs. 1 μM in rat) to amplify responses mediated by the α7 nAChR. Furthermore, although the ACh responses in the presence of PNU were specifically inhibited by ArIB[V11L;V16D] in both mouse and rat, the affinity of the peptide for the mouse α7 nAChR was apparently lower than for rat (incomplete block in mouse), and more rapidly reversible (**Figures 2** and **3**). These relatively minor pharmacological differences do not compromise the conclusion that the nAChR subtypes expressed in DRG neurons are the same in both species.

Within the four broad categories of neurons defined by this study, diverse response phenotypes were observed in each. For example, following an ACh pulse (prior to application of PNU), some neurons responded with relatively rapid calcium decay kinetics (sharp peaks; e.g., **Figure 2A**, top two traces), while other cells demonstrated relatively slow calcium decay kinetics (broad peaks) with distinctive shoulders on each peak (e.g., **Figure 2A**, third and fourth traces from top). When a combination of different VGCC antagonists was applied to the bath, only the sharp ACh-elicited peak profile was observed prior to PNU application (see **Figure 2B**, top three traces), presumably because the Ca^2^^+^entry through VGCCs triggered by ACh was suppressed. These results indicate that in some neurons of the β4-expressing subclass, opening of nAChRs triggers a significant activation of VGCCs, which results in the broad peaks with shoulders. The diversity of these responses may represent differences in how nAChRs are functionally coupled to other signaling components in each neuronal subclass, and may reflect different expression levels and/or subtypes of VGCCs co-expressed with the nicotinic receptors. Such differences may correlate with other functional characters.

Additional phenotypic diversity was demonstrated by the apparent amplification of the ACh responses in the presence of PNU, in a subset of neurons (e.g., **Figure 2B**, bottom trace). A co-application of both VGCC antagonists and an antagonist of the mitochondrial Na^+^/Ca^2^^+^ exchanger was required to sharpen the ACh-elicted peaks in the presence of PNU, mediated by α7 nAChRs (**Figures 2** and **3**); the addition of VGCC antagonists alone was insufficient to sharpen the [Ca^2+^]_i_ response profile. These observations suggest that there is diversity in the functional coupling between nAChRs, VGCCs, and the mitochondrial Ca^2^^+^ transport that depends both on the identity of the nAChR subtypes, and presumably on which type of neuron those nAChR subtypes are found. These diverse phenotypic responses may ultimately prove useful for a more refined definition of rat and mouse DRG neuronal subclasses. Our approach enables us to explore functional links between individual signaling proteins in a cellular context, which has the potential to better integrate the rapid advances in molecular neuroscience to the cellular and systems levels.

Functional coupling between nAChRs and other receptors and ion channels has been shown previously. Interestingly, in multiple tissues and expression systems nAChRs that contain α3 or β2 subunits have been shown to be functionally coupled to the activation of VGCCs, whereas α7 nAChRs have been shown to be functionally coupled to both VGCCs and mechanisms of intracellular calcium release (Dajas-Bailador et al., 2002; Dajas-Bailador and Wonnacott, 2004; Dickinson et al., 2007; Shen and Yakel, 2009). Those results seem consistent with our data in DRG neurons, which suggest that the α3β4/α6β4 nAChRs are functionally coupled to VGCCs, but the α7 nAChRs are functionally coupled to both VGCCs and mitochondrial Ca^2^^+^ transport into the cytoplasm. In the rat pheochromocytoma cell line (PC12), non-α7 nAChRs were shown to be functionally coupled to L-type VGCCs (Dickinson et al., 2007). In rat pituitary cells (GH3) α7 nAChRs were shown to be functionally coupled to L-type VGCCs (Feuerbach et al., 2005). Various nAChR subtypes, VGCC subtypes and mitochondrial Ca^2^^+^ transport may be functionally coupled in specific DRG neuronal subclasses to regulate cytoplasmic Ca^2^^+^ concentration, which is a common endpoint of almost all signaling in the nervous system, regulating diverse processes from neurotransmitter release to gene-expression changes (Hille, 2001; Shen and Yakel, 2009). Among our future directions is a plan to explore the functional coupling between nAChRs and other signaling components within specific DRG neuronal subclasses in more detail.

There are a plethora of generalizations about the mammalian nervous system based on studies using mice and rats as model systems. This study provides a molecular assessment of divergent cell types at a population level between these two species, demonstrating clear similarities, but also important species differences. Clearly some caution needs to be exercised with respect to the standard practice of using mouse as a molecular genetic system, and rat for physiological studies. An isolated study from either species could easily be over-interpreted in its breadth of applicability across mammalian species or in its translational value.

## Conflict of Interest Statement

The authors declare that the research was conducted in the absence of any commercial or financial relationships that could be construed as a potential conflict of interest.

## Author Contributions

Arik J. Hone, Baldomero M. Olivera, and Russell W. Teichert designed research. Nathan J. Smith, Tosifa Memon, Simon Bossi, Thomas E. Smith, and Russell W. Teichert conducted research. Nathan J. Smith, Arik J. Hone, Tosifa Memon, Simon Bossi, Thomas E. Smith, Baldomero M. Olivera, and Russell W. Teichert analyzed data. J. Michael McIntosh, Baldomero M. Olivera, and Russell W. Teichert wrote the paper.
